# Increased PD-L1 expression in erlotinib-resistant NSCLC cells with *MET* gene amplification is reversed upon MET-TKI treatment

**DOI:** 10.18632/oncotarget.19920

**Published:** 2017-08-04

**Authors:** Christina Demuth, Morten Nørgaard Andersen, Kristine Raaby Jakobsen, Anne Tranberg Madsen, Boe Sandahl Sørensen

**Affiliations:** ^1^ Department of Clinical Biochemistry, Aarhus University Hospital, DK-8200 Aarhus N, Denmark; ^2^ Department of Biomedicine, Aarhus University, DK-8000 Aarhus C, Denmark

**Keywords:** erlotinib, resistance, NSCLC, PD-L1, MET

## Abstract

**Introduction:**

Cancer cells can achieve immune evasion by expressing the programmed death receptor 1 ligand (PD-L1) on the cell surface. Blockade of the receptor (PD-1) can avert this evasion. Here we aim at investigating PD-L1 expression in erlotinib-resistant lung cancer cells with *MET proto-oncogene* (*MET*) gene amplification.

**Materials and Methods:**

We employed an erlotinib-resistant NSCLC cell line with *MET* gene amplification. PD-L1 mRNA (qPCR) and protein (flow cytometry) expression was investigated after treatment with MET and mitogen-activated protein kinase (MAPK) targeting drugs (crizotinib and SCH772984, respectively).

**Results:**

We demonstrate that PD-L1 expression is increased in erlotinib-resistant non-small cell lung cancer (NSCLC) cells with *MET* gene amplification. Targeted inhibition of MET significantly decreases both gene and protein expression of PD-L1. Further, we demonstrate that inhibiting MAPK also results in a significant decrease in PD-L1 expression. Taken together these results show that expression of PD-L1 in the erlotinib-resistant cell line is associated with MET activity, and the downstream MAPK pathway.

**Conclusions:**

Our results demonstrate that PD-L1 expression is increased in erlotinib resistant NSCLC cells with *MET* gene amplification and that the increase can be averted by targeted inhibition of MET.

## INTRODUCTION

Worldwide, lung cancer is one of the most frequent cancers and mortality is high. The vast majority of lung cancers are non-small cell lung cancers (NSCLC). Over the past decade several oncogenic drivers have been identified [1], though most distinct are the observations of activating mutations in the epidermal growth factor receptor (*EGFR*) of patients with adenocarcinoma histology. Today, treatment with EGFR-targeting tyrosine kinase inhibitors (TKIs), like gefitinib and erlotinib, is part of the routine treatment of patients harboring these mutations. This change in treatment has markedly increased the survival in this subgroup of patients [2], though treatment resistance is inescapable [3]. Several resistance mechanisms have been found and thoroughly described including the *EGFR* T790M mutation, *MET proto-oncogene* (*MET*) gene amplification, development of epithelial to mesenchymal transition (EMT), and transition to a small cell lung cancer (SCLC) phenotype [4].

Treatment of lung cancer with immunotherapy has gained much attention after promising clinical trials. Especially blockade of the pathway activated by the programmed death receptor 1 (PD-1) and its ligand PD-L1 is being massively investigated. PD-1 is expressed on the surface of T-cells, and upon activation by the ligand, PD-1 initiates inactivation of the cell [5]. Expression of PD-L1 on the surface of cancer cells leads to this inactivation of T-cells and helps the cancer cells achieve immune evasion [6]. The T-cell inactivating interaction can be inhibited by anti-PD-1 or anti-PD-L1 agents, and hereby anti-tumor immune activity can be restored [7, 8]. At present, no validated biomarker for response to PD-1-PD-L1 blockade has been presented [9], though studies suggest that expression of PD-L1 in tumor specimens could be a candidate biomarker [10, 11].

Studies of PD-L1 expression have shown an association with activating *EGFR* mutations in NSCLC [12–14]. Though, contradicting results have also been presented [15]. Activation of EGFR increases the expression of PD-L1 *in vitro* through the mitogen-activated protein kinase (MAPK) pathway, and inhibition of EGFR using a TKI correspondingly decreases expression of PD-L1 [16–18]. This regulatory mechanism has been confirmed *in vivo* [19]. Despite increasing interest in the mechanisms of resistance to EGFR-TKIs, only few studies have engaged in investigating the expression of PD-L1, when resistance has emerged. *In vitro* data suggest that the T790M resistance mutation is accompanied by increased expression of *PD-L1* [16], and a retrospective clinical study finds association between gefitinib resistance and increased PD-L1 expression in a patient cohort [20]. If the dynamics of PD-L1 are affected by treatment and development of resistance, the timing of retrieving the biopsy used for investigating PD-L1 expression is of great importance.

The aim of this study was to investigate expression of *PD-L1* in erlotinib-resistant cells. The cells acquired resistance through *MET* gene amplification, and we further wanted to investigate if targeting the new oncogenic driver and the downstream proliferative pathway would affect the *PD-L1* expression.

## RESULTS

### PD-L1 expression in erlotinib-resistant cells

The erlotinib-resistant cell line HCC827ER was generated over approximately 4 months where the erlotinib concentration was gradually increased to a maximal concentration of 5 μM. A full description of the resistant cells is presented in Jakobsen et al. [21].

**Figure 1:**
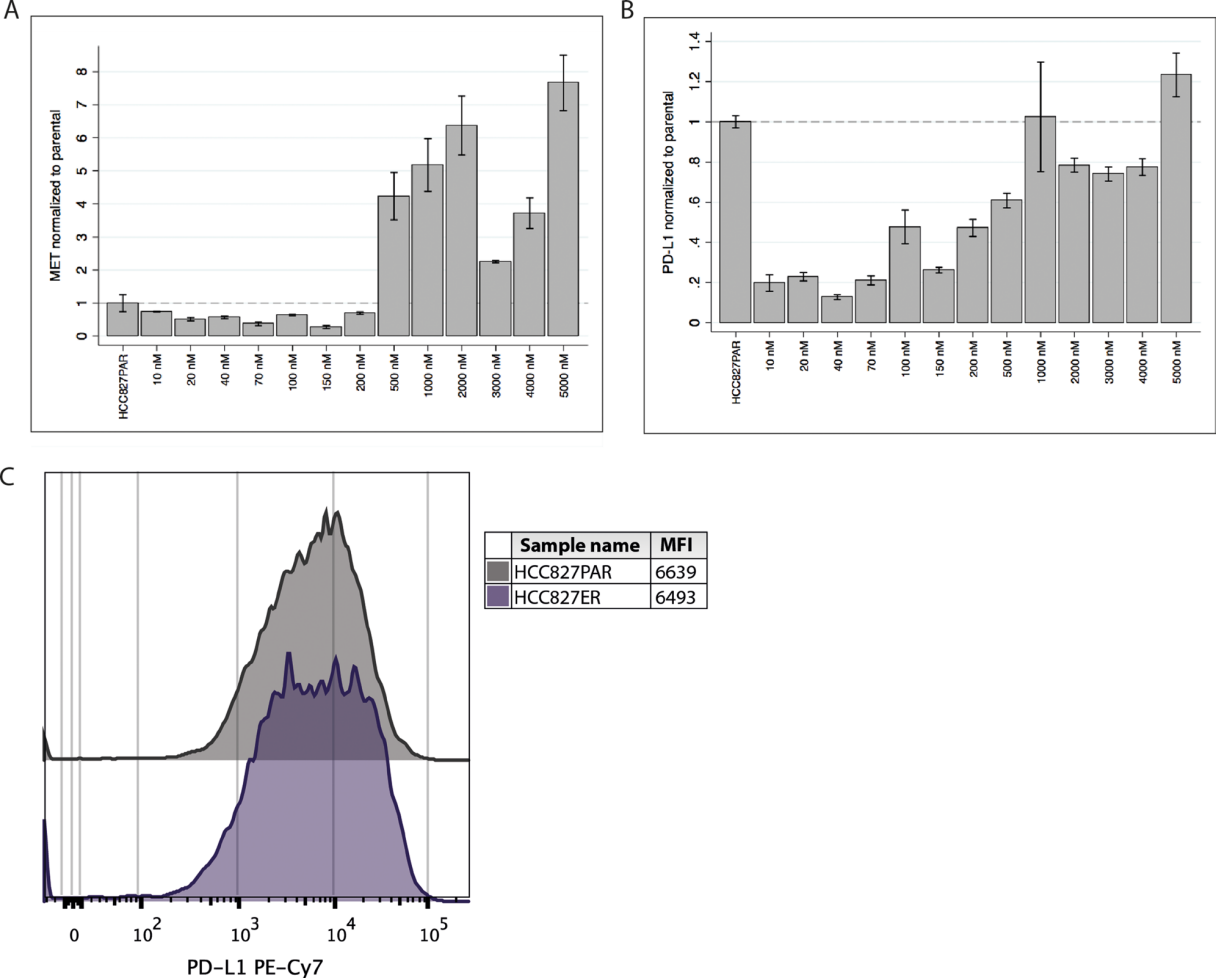
*PD-L1* and *MET* gene expression and PD-L1 cell surface expression in HCC827ER (**A**) *MET* gene expression was measured at each concentration-point during establishment of the resistant cell line (the erlotinib concentrations are indicated at the x-axis), and normalized to the expression in the HCC827PAR cell line. Initially expression is decreased, but during resistance development expression starts increasing (approximately from 200 nM erlotinib), and markedly exceeds that level of the HCC827PAR when resistance is established. (**B**) Correspondingly, *PD-L1* gene expression was measured during the resistance-development, and normalized to HCC827PAR. *PD-L1* expression also starts increasing at approximately 200 nM erlotinib, and expression in the final erlotinib-resistant cell line exceeds that of HCC827PAR. (**C**) Using flow cytometry the PD-L1 protein expression was measured. A representative histogram and mean fluorescence intensity (MFI) values are presented.

We investigated the gene expression of *MET* and *PD-L1* by qPCR at each erlotinib concentration during the establishment of HCC827ER. We observed that *MET* gene expression initially decreased but started increasing at approximately 200 nM (see Figure 1A). At resistance the *MET* gene expression markedly surpassed that of the parental cell line. This increase followed the increase in gene copy number [21]. We observed a small decrease in *MET* gene expression at 3 and 4 μM erlotinib, although it still greatly surpassed that of the parental cell line. Interestingly, the gene expression of *PD-L1* seemed to follow that of *MET*. *PD-L1* gene expression was markedly decreased at the initiation of erlotinib treatment (see Figure 1B), but when the erlotinib concentration reached approximately 200 nM, expression of *PD-L1* started increasing and at the point of resistance (5 μM), *PD-L1* expression had exceeded the expression level of the parental cells.

We used flow cytometry to investigate the expression of PD-L1 protein on the surface of the cells. As can be seen from Figure 1C, the median fluorescence intensity (MFI) and histograms were similar between the parental HCC827PAR and the HCC827ER cell line. These results corroborated the findings of increased *PD-L1* gene expression.

### PD-L1 expression was decreased in HCC827ER upon treatment with crizotinib

Previous studies indicate a general link between receptor tyrosine kinase (RTK) activity and expression of *PD-L1* in erlotinib sensitive cells [16, 17, 22]. Our first results suggested that an association between increase in *MET* and *PD-L1* gene expression was also present in erlotinib-resistant cells. We therefore wanted to investigate if inhibition of MET, using a MET-targeting TKI (crizotinib), affected the expression of *PD-L1* in these cells.

**Figure 2:**
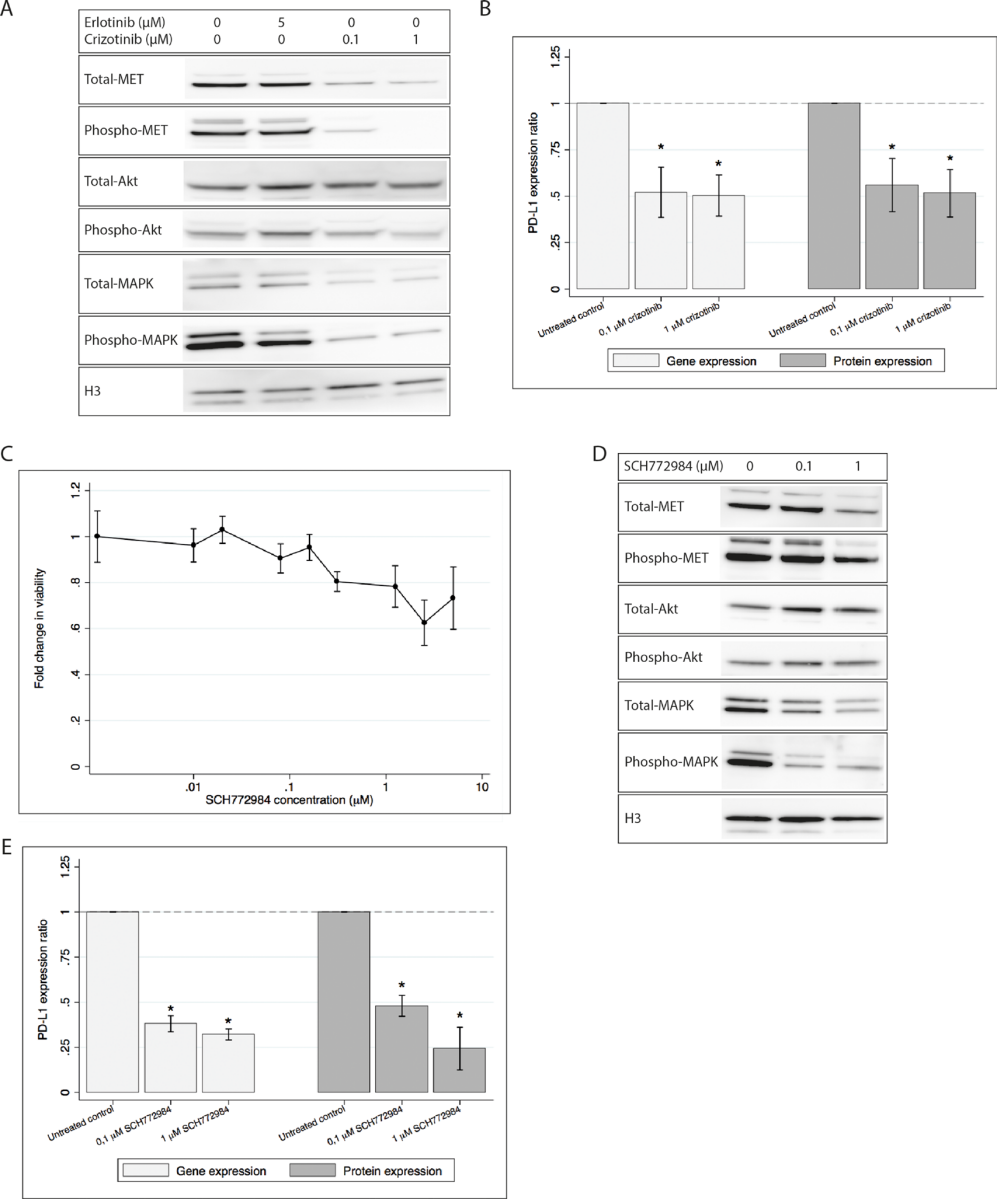
PD-L1 gene and protein expression is decreased in HCCC827ER upon treatment with either crizotinib or SCH779428 H3 is used as loading control in both western blots. All gene and protein expressions are normalized to an untreated control and presented as mean ± SD. Gene and cell surface protein expression data is presented as mean of three individual experiments. * denotes significant difference (*p*-value < 0.05) (**A**) Western blot showing decreased activation of MET, Akt and MAPK after treatment with crizotinib, while erlotinib shows only a minor effect on MAPK. (**B**) PD-L1 gene and protein expression after treatment with crizotinib. Treatment with crizotinib significantly decreases the expression of PD-L1. (**C**) Cell viability after treatment with SCH772984. Viability measurements are normalized to an untreated control, and the fold change is plotted. (**D**) Western blot showing decreased MET, Akt and MAPK activity after treatment with SCH772984. (**E**) PD-L1 gene and protein expression following treatment with SCH772984. Gene expression and protein expression is significantly decreased by treatment with SCH772984.

The viability of HCC827ER cells following treatment with crizotinib has previously been determined [21]. We found that treatment with 0.1 and 1 μM crizotinib sustained reasonable viability, while sufficiently inhibiting phosphorylation of MET (Figure 2A). A decrease in total-MET was also observed, though not as distinct as the decrease in phospho-MET. Furthermore, the downstream phosphorylation of Akt and MAPK was also decreased by crizotinib (see Figure 2A). Treatment with both 0.1 and 1 μM crizotinib significantly decreased gene and protein expression of PD-L1 (Figure 2B) as measured by qPCR and flow cytometry.

5 μM erlotinib showed a tendency towards decreasing PD-L1 gene expression (data not shown), but this effect was not significant and neither was the trend seen on the protein expression (see Supplementary Figure 2). Combined treatment using both crizotinib and erlotinib had no additional effect on either cell viability or PD-L1 expression (data not shown).

These results demonstrated that inhibiting MET using crizotinib resulted in decreased PD-L1 expression.

### PD-L1 expression is decreased upon MAPK inhibition

Since the MAPK pathway was activated by the *MET* gene amplification, and we saw that crizotinib inhibited phosphorylation of MAPK, we wanted to investigate if PD-L1 expression in HCC827ER was dependent on the MAPK pathway. Cells were treated with the MAPK inhibitor SCH772984. Cell viability was only mildly affected by the inhibitor at concentrations up to 5 μM (see Figure 2C). Different concentrations of SCH772984 were tested and we found that 0.1 and 1 μM SCH772984 was sufficient for decreasing MAPK activity (see Figure 2D). Total and activated Akt were stable or only weakly increased upon treatment.

After exposure to SCH772984 the HCC827ER cell line significantly decreased expression of *PD-L1* mRNA (see Figure 2E), as measured by qPCR. Further, PD-L1 cell surface expression was investigated by flow cytometry, and we found PD-L1 to be significantly decreased in treated cells as compared to the untreated control (see Figure 2E).

These results indicated that inhibiting MAPK led to decreased levels of PD-L1.

### PD-L1 expression in HCC827ER subclones

The erlotinib resistant cell line can be divided into either *MET*-amplified or EMT subclones [21]. This polyclonality was also visualized in the flow cytometry histogram, where the HCC827ER cell line presented with a broad histogram and a tendency of two peaks, indicating clonal populations with either high or low expression of PD-L1 (Supplementary Figure 2).

**Figure 3:**
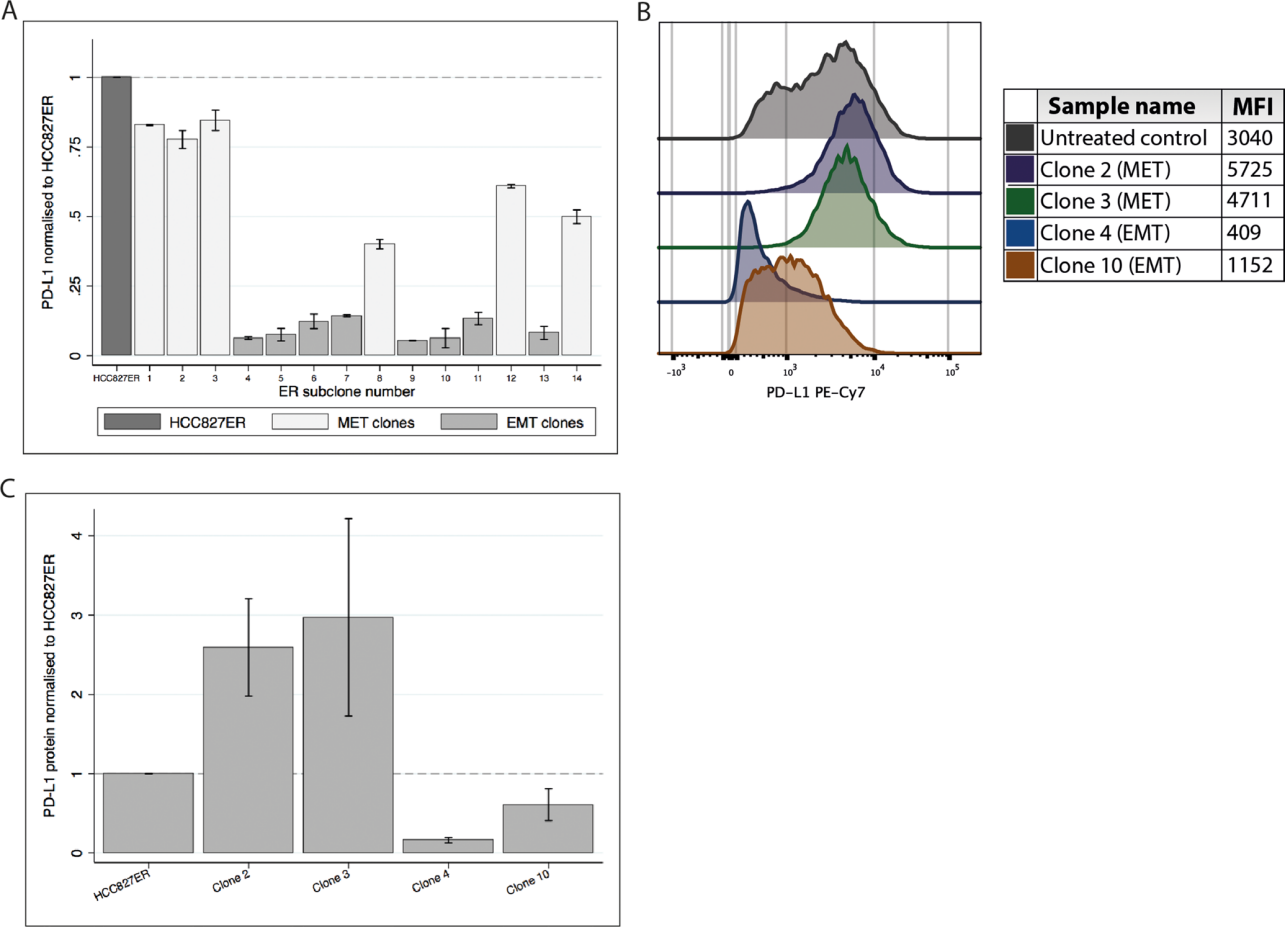
PD-L1 gene and protein expression in HCC827ER sub clones (**A**) *PD-L1* gene expression in the 14 clones (normalized to HCC827ER). Expression of *PD-L1* is markedly higher in the MET clones compared to the EMT clones. (**B**) Representable histogram from a flow cytometry analysis of HCC827ER and four clones. MFI values are found in the table. (**C**) A bar graph showing the mean and SD of PD-L1 protein expression collected from three individual experiments.

We wanted to investigate how PD-L1 expression differed in these clonal subtypes. We measured *PD-L1* gene expression in 14 sub clones derived from HCC827ER (Figure 3A). There was a clear distinction between the two clonal subtypes; *MET*-amplified clones generally presented with higher *PD-L1* expression than the EMT clones. Further, we investigated the protein expression using flow cytometry on four of the 14 clones (two MET and two EMT clones, Figure 3B and 3C). The *MET*-amplified clones (clone 2 and 3) had a pronounced increase in PD-L1 protein expression as compared to the original HCC827ER cell line, while the opposite was the case for the EMT clones (clone 4 and 10).

These results further strengthened the hypothesis of a correlation between MET activity and increased PD-L1 expression, while PD-L1 expression seemed to be lowered in EMT clones.

## DISCUSSION

Immune evasion is one of the hallmarks of cancer [23]. One way of evading the immune system is by expressing PD-L1 on the surface of the cancer cell. This evasion can be targeted by immunotherapy, which is currently among the most promising new treatments of NSCLC. As of today the primary suggested biomarker for response to PD1/PD-L1 system blockade is expression of PD-L1. Further, studies have correlated expression of PD-L1 to *EGFR* mutational status and *in vitro* data have confirmed a regulatory function of EGFR [16, 17]. Though, not much is known about the dynamics in PD-L1 expression when EGFR-TKI resistance occurs. In the present study, we have investigated expression of PD-L1 during development of erlotinib-resistance in the NSCLC cell line HCC827. The resistant cell line was generated using a clinically relevant erlotinib concentration [24]. We found that the expression is diminished initially, but levels exceed expression in the parental cell line, when resistance has developed. The resistant cells gain gene expression of the new oncogenic driver *MET* and we further demonstrate that expression of PD-L1 increases as expression of *MET* increases. Treatment with the MET-directed TKI crizotinib decreases expression of *PD-L1*. Further, targeting MAPK, downstream in the signaling pathway of MET, also effectively decreases expression of PD-L1. These results suggest an association between MET activity and PD-L1 expression.

Our results are in accordance with previous findings of RTK regulated PD-L1 expression and its involvement with MAPK [16–18, 22, 25]. Taken together, these studies indicate a general regulatory mechanism of RTKs through the MAPK pathway, and in some cases, through the PI3K/Akt pathway or a combination of the two [22, 26]. If PD-L1 expression is validated as a biomarker for PD1-PD-L1 axis blockade, this makes TKI-resistance mediated by activation of the MAPK or PI3K/Akt pathways (by-pass mechanisms) extraordinarily interesting in regard to immunotherapy, both when it comes to second line treatment of patients and the use of PD-L1 as biomarker of response. Jiang and coworkers have already presented data showing that BRAF inhibitor resistant melanoma cell lines increase the expression of PD-L1 using the PI3K-STAT3 pathway [27]. Further, a study comparing tumor PD-L1 expression in biopsies taken before initiation of gefitinib and at resistance found increased expression in 7 of 18 patients [20]. They also found that the increase in PD-L1 expression was associated with MET positivity (as measured by immunohistochemistry). The authors note that the number of patients included in this study is too low to make general conclusions, though in the context of ours and other studies, the biological evidence seems to back this clinical finding. However, for full disclosure on the biological mechanism, further studies are needed. *In vitro* RNAi studies and/or *in vivo* models should be used to investigate expression of PD-L1 in *MET* knock-out models to present evidence of MET-regulated PD-L1 expression. Furthermore, the biological effect of the increase in PD-L1 expression should be investigated in the presence of a functional immune system.

The erlotinib-resistant cell line investigated in the present study consists of subclones that can be divided into *MET*-amplified and EMT clones [21]. When investigating the clones separately we found that the *MET*-amplified clones have a markedly higher expression of PD-L1 than does the EMT clones. Lack of induced PD-L1 expression in EMT clones contradicts previous research on the subject. Generally studies have shown that the mesenchymal phenotype (intrinsic or induced by EMT) is associated with increased PD-L1 expression in different cancers [28–32]. In lung cancer specifically, Chen and colleagues have shown that PD-L1 expression is regulated by the miR-200/ZEB1 loop, and suggest that increased expression of ZEB1 in mesenchymal cell lines prompt increased expression of PD-L1. Despite increased ZEB1 expression in our resistant EMT clones [21], we did not observe increased PD-L1 expression. Further, Kurimoto and colleagues find that induction of EMT in HCC827 results in increased expression of PD-L1 [29]. A possibility could be that induction of EMT by TGF-β and FGF2 used in that study, results in a different phenotype than induction by acquired erlotinib resistance.

The data presented here suggest that erlotinib-resistant cells increase the expression of PD-L1 through a mechanism involving MET and MAPK activation. Treatment with MET inhibitors in relevant cases could possibly prevent PD-L1 expression and thereby immune evasion. Furthermore, the PD-L1 fluctuations observed here, urges clinicians to exercise caution when evaluating the use of immunotherapy after TKI-treatment, based on PD-L1 measurements on biopsies taken at the time of diagnosis.

## MATERIALS AND METHODS

### Cell lines and treatment

The cell line HCC827 was purchased from ATCC. An erlotinib-resistant HCC827 cell line (HCC827ER) was generated as previously described [21]. Further, HCC827ER clones were established using minimal dilution [21]. All cells were maintained in RPMI 1640 media supplemented with 1% Penicillin-Streptomycin (Gibco), 1% Hepes 1 M buffer solution (Gibco), 1% Sodium Pyruvate (Gibco), 10% Fetal Bovine Serum (Gibco), and 1% 250 μg/mL Amphotericin B solution (Sigma-Aldrich). Further, the resistant cell line and clones were maintained in 5 μM erlotinib (Selleckchem).

For all mRNA and flow cytometry experiments cells were plated in 6-well plates and incubated for 24 h (300,000 cells/well). After incubation the cells were treated with drug and incubated for 72 h prior to harvest.

The inhibitors erlotinib, crizotinib, and SCH772984 were all purchased from Selleckchem.

### Viability

For cell viability studies cells were plated in 96-well plates (5000 cells/well) and incubated for 24 h. Cells were treated with drug as indicated and incubated for 72 h prior to viability assessment. Cell viability was tested using an MTS assay (CellTiter 96^®^ AQueous Non-Radioactive Cell Proliferation Assay, Promega) following the manufacturer's instructions. Colorimetric measurement was performed using a Multiscan Ascent plate reader (Thermo Electron Corporation).

### Western blotting

Protein was harvested using Lysis Buffer 17 (R&D Systems) supplemented with 10 μg/mL of each inhibitor Pepstatin, Leupeptin, and Aprotinin. Harvest was performed according to manufacturer's protocol. The protein concentration was determined using the Qubit^®^ 2.0 Fluorometer (Thermo Scientific). 30 μg protein was resolved on 4–12% Bis-Tris gels (Life Technologies) and transferred to a PVDF membrane (Thermo Scientific). Membranes were blocked in 1X TBST with 5% non-fat dry milk (protein-specific antibodies) or 5% BSA (phospho-specific antibodies). For detection the following primary antibodies and dilutions were used: anti-EGFR (Abcam, 1:1000), anti-EGFR phospho-Tyr1173 (LSBio, 1:500), anti-Akt (Cell Signaling Technology, 1:500), anti-Akt phospho-Ser473 (Cell Signaling Technology, 1:500), anti-MAPK (Cell Signaling Technology, 1:1000), anti-MAPK phospho-Thr202/Tyr204 (Cell Signaling Technology, 1:1000), and anti-Histone H3 (Cell Signaling Technology, 1:2000). Antibodies were diluted in either 1X TBST with 5% non-fat dry milk or 5% BSA corresponding to the blocking agent. Goat anti-mouse (DAKO, 1:4000) and goat anti-rabbit secondary antibody (Cell Signaling Technology, 1:5000) were diluted in 1X TBST with 5% non-fat dry milk and used for detection of total-MET and the remaining proteins, respectively. The membranes were added SuperSignal Dura West Chemiluminescent Substrate (ECL) (Thermo Scientific) followed by development in an ImageQuant LAS 4000 scanner (GE Healthcare Life Sciences).

### cDNA synthesis and quantitative PCR

For RNA analysis cells were harvested using Nunc Cell Scrapers (Thermo Scientific), centrifuged, and resuspended in 350 μL RLT buffer (Qiagen). Total RNA was extracted using RNeasy mini kit on a QIACube instrument (Qiagen). RNA concentration was determined using the NanoDrop 2000 (Thermo Fisher Scientific). 200 ng RNA was used for cDNA synthesis. cDNA was synthesised in a 20 μL reaction consisting of 2.5 μM Oligo(dT) (DNA Technology), 1 mM of each dNTP (VWR), 2.5 units/μL MulV Reverse Transcriptase, 1 units/μL RNase inhibitors, 1xPCR buffer, and 6.25 mM MgCL2 (all from Applied Biosystems). Quantitative PCR (qPCR) was performed using LightCycler 480 SYBR Green I Master on the LightCycler 480 platform (Roche).

Five reference genes (β-actin, HMBS, GAPDH, YWHAZ and B2M) were tested and compared using the Normfinder Software [33]. Among these β-actin (ACTB) was found most stably expressed in the cell lines and was used as reference gene for the following experiments.

For the experiments presented the following primer sequences, concentrations and annealing temperatures were applied:

ACTB: forward 5′- GGCGCCACCACCATGTA CCCT-3′, reverse 5′- AGGGGCCGGACTCGTCATACT-3′, 0.25 μM, 68°C.

PD-L1: forward 5′- GGTGGTGCCGACTACAA GCGA-3′, reverse 5′-TGACTTCGGCCTTGGGGTAGC-3′, 0.25 μM, 64°C.

MET: forward 5′- TGGAGACACTGGATGGGAGT-3′, reverse 5′-CAGCGCGTTGACTTATTCAT-3′, 0.25 μM, 60°C.

All primers were purchased from Eurofins Genomics. Gene expression was measured and calculated using the Lightcycler 480 instrument (Roche) and the second derivative max method using standard curves.

All experiments were performed in biological triplicates, except from the analysis of RNA from the resistance development (Figure 1A and 1B) and from the 14 sub-clones (Figure 3A), where only one specimen was available. All gene expression analyses were performed in technical triplicates.

### Flow cytometry

Cells were detached from wells using PBS/0.5% BSA/2 mM EDTA, washed once in staining buffer (PBS/0.5% BSA/0.09% sodium azide), and kept on ice. Non-specific antibody binding was blocked with 10% mouse serum (from healthy C57BL/6J mice) for 15min at 4°C. Samples were then stained with Live/Dead fixable dye near-IR (Life Technologies) and mouse anti-human PD-L1 PE-Cy7 (0.5 g/mL, clone 29E.2A3, BioLegend) in stain buffer for 30 min (in the dark at 4°C). Cells were washed in stain buffer, fixed in PBS/0.9% formaldehyde (Sigma-Aldrich), and analysed immediately on an LSR Fortessa flow cytometer (BD Biosciences).

Compensation was done using single stained beads, for PD-L1 PE-Cy7 Comp Beads Plus (BD Biosciences) were used and for Live/Dead nIR ArC Amine Reactive Compensation beads (Life Technologies) were used. Data was analysed and figures were made using FlowJo 10.0.7 for Mac (FlowJo, LLC). For gating strategy, see Supplementary Figure 1.

All flow cytometry experiments were performed using the LSR Fortessa flow cytometer at the FACS Core Facility, Aarhus University, Denmark.

### Graphs and statistics

All graphs and statistics are produced in Stata 13 (StataCorp, 2013).

Gene and protein expression data from TKI experiments is collected from three individual setups. In each experiment data was normalised to an untreated control, and the mean of this normalised data is presented in graphs.

All data is presented as mean ± standard deviation (SD) (mean of biological triplicates). The two-tailed Student's *t*-test was used to assess the difference between two groups, and *p*-values < 0.05 were considered significant.

## SUPPLEMENTARY MATERIALS FIGURES


