# Supplementary material for: Increased PD-L1 expression in erlotinib-resistant NSCLC cells with MET gene amplification is reversed upon MET-TKI treatment
Source: Oncotarget. 2017 Aug 4;8(40):68221–9. doi: 10.18632/oncotarget.19920 (PMC5620250; doi:10.18632/oncotarget.19920)
Supplement: Supplementary file 1 [file oncotarget-08-68221-s001.pdf]

Increased PD-L1 expression in erlotinib-resistant NSCLC cells with *MET* gene amplification is reversed upon MET-TKI treatment

SUPPLEMENTARY MATERIALS

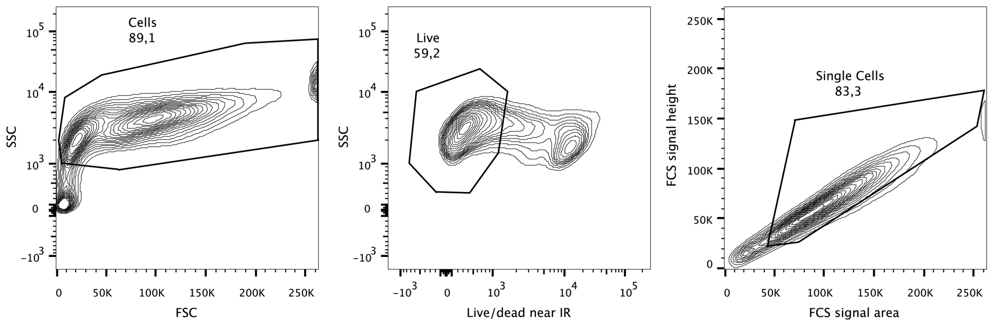

Supplementary Figure 1: The gating strategy used in the present study is shown on representative plots. First, debris was excluded on a forward scatter (FSC) vs. side scatter plot. Live cells were then identified as Live/dead nIR negative events, and doublets were excluded on a plot of FSC-area vs. FCS-height signals. Events in the gate containing live/single cells were used in further analyses.

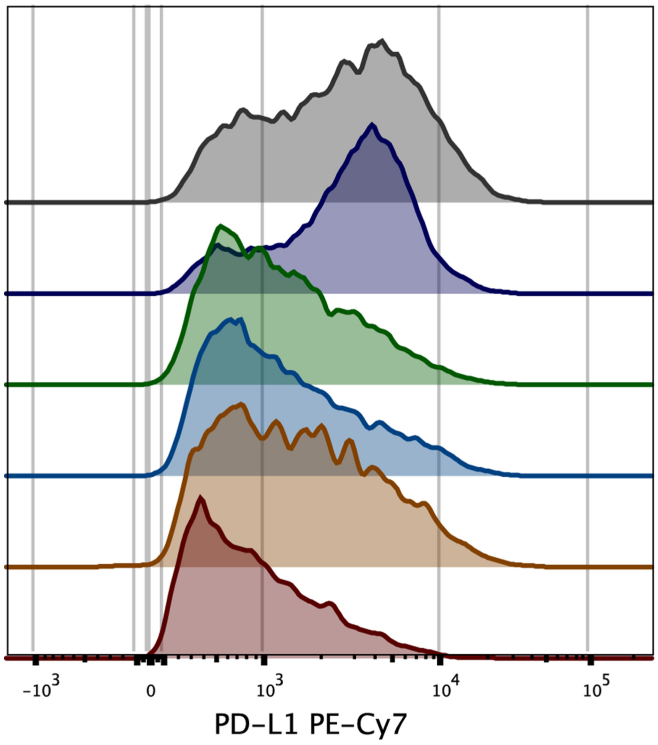

	Sample name	MFI
	Untreated control	3040
	5 μM erlotinib	3339
	0.1 μM crizotinib	1199
	1 μM crizotinib	1177
	0.1 μM SCH772984	1532
	1 μM SCH772984	819

Supplementary Figure 2: A representative histogram and table with MFI values from one flow cytometry analysis. HCC827ER cells are either left untreated or treated with erlotinib, crizotinib or SCH772984.
